# Supplementary material for: TAS2R38 Genotype Does Not Affect SARS-CoV-2 Infection in Primary Ciliary Dyskinesia
Source: Int J Mol Sci. 2024 Aug 8;25(16):8635. doi: 10.3390/ijms25168635 (PMC11354733; doi:10.3390/ijms25168635)
Supplement: Supplementary file 1 [file ijms-25-08635-s001.zip › Supplementary file 1.pdf]

Clinical symptoms in PCD COVID-acquired patients as reported on ARTIQ questionnaire (percentage and 95 % CI)

Taste/smell reduction	8.33 (1.49-35.4)
Increased cough	41.7 (19.3-68.1)
Hearing loss	0
Blocked nose	50 (25.4-74.6)
Runny nose	58.3 (31.9-80.7)
Sneezing	25 (8.9-53.2)
Lacrimation	0
Raucousness	0
Fever	66.7 (39.1-86.2)
Swelling	0
Chills	0
Headache	25 (8.9-53.2)
Sore throath	16.7 (4.7-44.8)
Muscle or joint pains	41.7 (19.3-68.1)
Chest pain	0
Sinonasal pain	33.3 (13.8-60.9)
Neck tumefaction	0
Problems of breathing	16.7 (4.7-44.8)
Dyspnea	25 (8.9-53.2)
Asthenia	83.3 (55.2-95.3)
Loss of appetite	0
Diarrhea	0
Nausea	0
Vomiting	0
Abdominal pain	0
Dizziness	0
Poor quality of sleep	0
Difficulty in concentration	0
