# Supplementary figures and images for: TAS2R38 Genotype Does Not Affect SARS-CoV-2 Infection in Primary Ciliary Dyskinesia
Source: Int J Mol Sci. 2024 Aug 8;25(16):8635. doi: 10.3390/ijms25168635 (PMC11354733; doi:10.3390/ijms25168635)

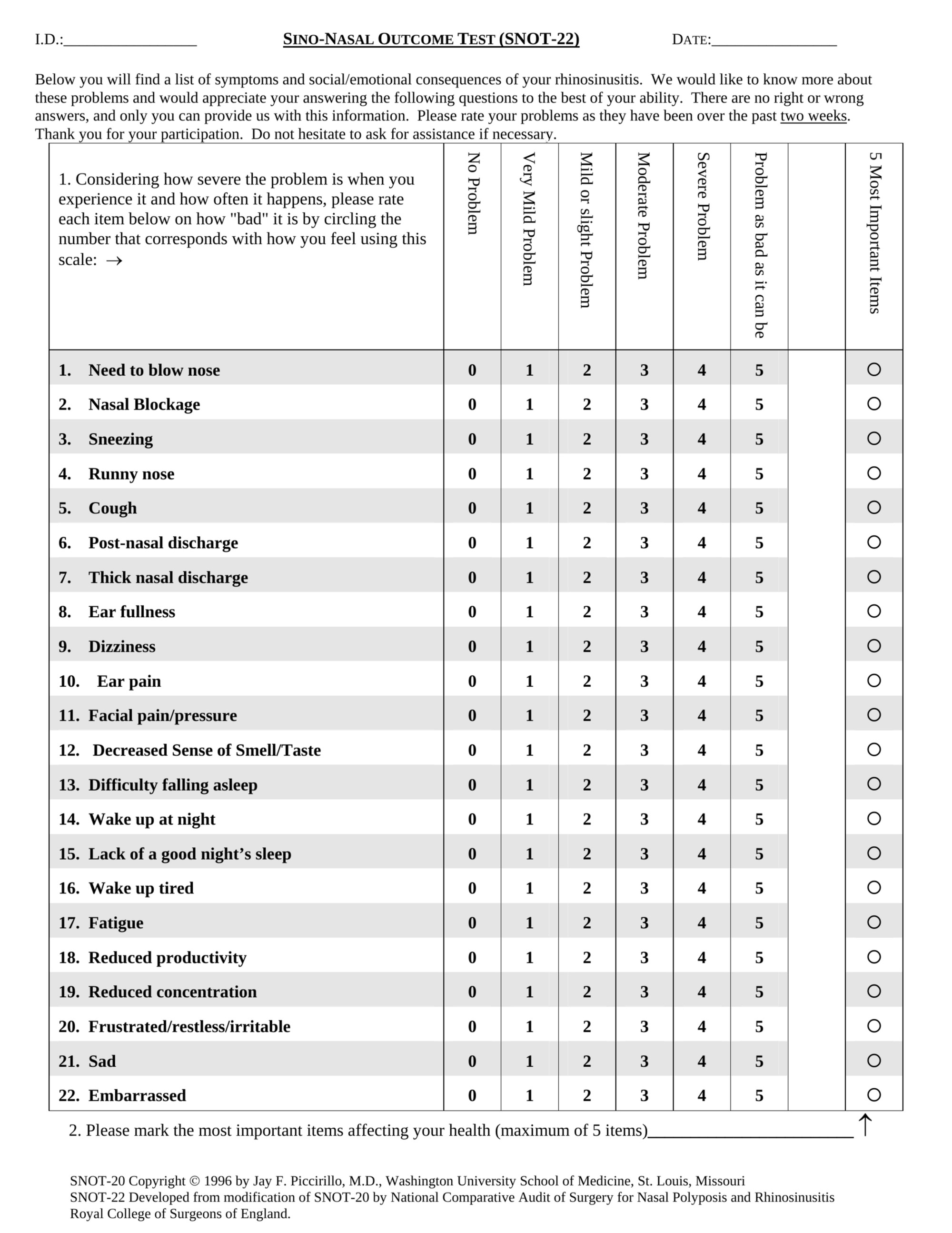

Supplement: Supplementary file 1 [file ijms-25-08635-s001.zip › Supplemementary file 2 SNOT-22 english.jpeg]
